# Strategies for genetic manipulation of adult stem cell-derived organoids

**DOI:** 10.1038/s12276-021-00609-8

**Published:** 2021-10-18

**Authors:** Constantin Menche, Henner F. Farin

**Affiliations:** 1 Georg-Speyer-Haus, Institute for Tumor Biology and Experimental Therapy, Frankfurt am Main, Germany; 2 Frankfurt Cancer Institute, Goethe University, Frankfurt am Main, Germany; 3 German Cancer Consortium (DKTK), Heidelberg, Germany; 4 German Cancer Research Center (DKFZ), Heidelberg, Germany

**Keywords:** Mechanisms of disease, Genetic engineering, Mutagenesis, Experimental models of disease

## Abstract

Organoid technology allows the expansion of primary epithelial cells from normal and diseased tissues, providing a unique model for human (patho)biology. In a three-dimensional environment, adult stem cells self-organize and differentiate to gain tissue-specific features. Accessibility to genetic manipulation enables the investigation of the molecular mechanisms underlying cell fate regulation, cell differentiation and cell interactions. In recent years, powerful methodologies using lentiviral transgenesis, CRISPR/Cas9 gene editing, and single-cell readouts have been developed to study gene function and carry out genetic screens in organoids. However, the multicellularity and dynamic nature of stem cell-derived organoids also present challenges for genetic experimentation. In this review, we focus on adult gastrointestinal organoids and summarize the state-of-the-art protocols for successful transgenesis. We provide an outlook on emerging genetic techniques that could further increase the applicability of organoids and enhance the potential of organoid-based techniques to deepen our understanding of gene function in tissue biology.

## Organ model: Making custom 3D models using genetic manipulation

Advances in targeted genomic manipulation are expanding the utility of cultured organ models for studying disease in complex tissues. Mammalian cells can be coaxed to self-assemble and mature into organoids, structures that recapitulate key features and functions of various organs. Constantin Menche and Henner Farin from the Georg-Speyer-Haus, Institute for Tumor Biology and Experimental Therapy, Frankfurt am Main, Germany, have reviewed how technologies like the CRISPR/Cas9 genome editing system can be used to “customize” such organoid cultures. They highlight challenges associated with achieving efficient genetic manipulation in gastrointestinal organoid systems. These include delivering the necessary reagents for genome editing into cells, and ensuring that only the desired changes are made to the DNA. Under optimal conditions, researchers can test the tissue-level effects of deleting or altering genes, or study the impact of mutations that have been observed in patients.

## Introduction

Organoids are multicellular structures that form in the presence of a three-dimensional (3D) extracellular matrix (ECM) and defined growth factors^1,2^. Because the environment mirrors the physiologic environment in vitro, the cells self-organize, recapitulating the architectural features of the organ of origin. In comparison to classical cell lines, organoids offer two key advantages. First, stable primary cell cultures can be established without prior cell transformation or immortalization, thereby preserving the phenotypic characteristics. Second, the cultures retain the capacity to generate differentiated progeny, providing a system to study stem cell biology and physiological interactions among mature cell types. Since their inception a decade ago^3–5^, intestinal organoid models have revolutionized many aspects of biological research, including research in immunology, microbial interactions, and cancer biology^6–8^. Efficient protocols for the propagation of organoids have been established for both human and mouse tissues, including liver, pancreas, prostate, and lung tissues^9–15^. In addition to organoids propagated from normal tissues, biobanks of patient-derived tumor organoids (PDTOs) have been established, and these have become an important tool to replicate interindividual tumor heterogeneity and to test drug responses in vitro^16–19^. The combination of these tools with improved analytical methods in genomics, transcriptomics, proteomics, and metabolomics has provided new possibilities to study human physiology, and the technological advances in single-cell analysis and 3D live imaging allow dynamic investigation of cellular states. These new research opportunities clearly outweigh the fact that this experimentation is more costly, time-consuming, and technically demanding than traditional experiments in cell lines. An important consideration is that working with primary human cell models and associated data requires informed patient consent and adherence to institutional and governmental regulations^20^.

**Fig. 1:**
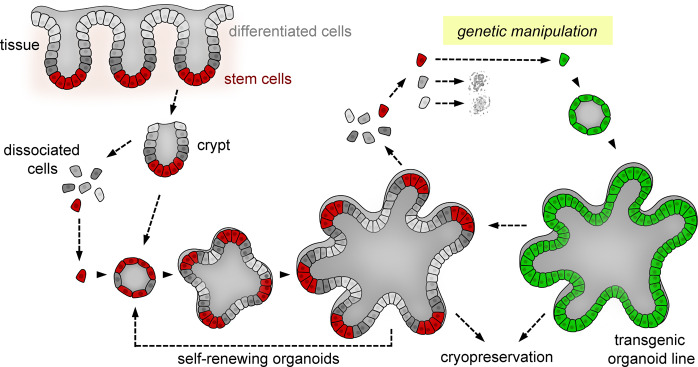
Organoids as a genetic model system. Organoid lines derived from the gastrointestinal epithelium can be generated from isolated crypts or single adult stem cells (red). Ex vivo, stem cells self-renew and generate differentiated progeny (gray). By targeting single stem cells, transgenic organoids that can be clonally expanded and cryopreserved (green) are derived. In contrast, differentiated cells cannot form new organoids.

The introduction of transgenes, reporter constructs, and targeted genetic alterations further expands the potential of organoid research. Several key features predestine organoids as a genetic model (Fig. 1). Long-term stable cultures can be established from single stem cells^3^, a method that facilitates the generation of clonal organoid lines. Furthermore, cryopreservation protocols that enable the establishment of “living” biobanks from large cohorts of individuals have been optimized^21^. Efficient methods to introduce foreign genetic material have been described, including transient transfection^22^, electroporation^23,24^ of plasmid DNA, or viral vector transduction^25,26^. The advent of CRISPR/Cas9 technology has provided vast possibilities for targeted genetic engineering of organoid models^27^. In primary cells from different individuals, the genetic background can significantly impact phenotypic features, and the possibility of generating isogenic controls allows variation from this source to be diminished^28^.

In this review, we focus on adult gastrointestinal organoids of mouse and human origin and summarize the state-of-the-art protocols for genetic manipulation. We emphasize the technical features of different methods to introduce transgenes, target specific loci, and characterize engineered organoid cells. Thereby, we aim to provide guidance for selecting the optimal approach and identifying the technical features that should be taken into consideration. Finally, we discuss the challenges and future opportunities for transgenesis using adult stem cell-derived organoid models.

## General considerations for genetic investigation of multicellular organoids

**Fig. 2:**
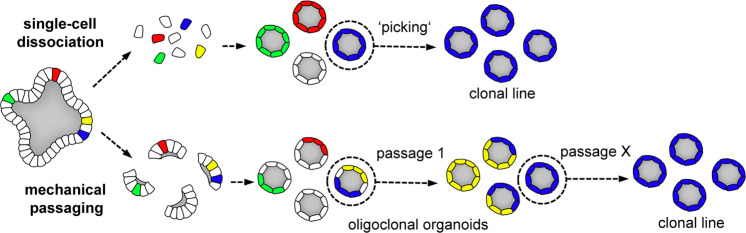
Stem cell dynamics in multicellular organoids. Self-renewal in organoids is driven by stem cells that multiply as the organoids grow. Independent stem cells and their progeny are labeled in different colors. The clonal composition of organoids after passaging depends on the procedure: culture after single-cell dissociation and expansion of single organoids (“picking”) allows the derivation of clonal lines, whereas mechanical dissociation preserves larger fragments that contain multiple stem cells, resulting in mosaics. During repeated passaging, the organoids progressively become monoclonal due to neutral drift.

As dynamic structures, gastrointestinal organoids reflect the epithelial turnover in the gut. In particular, mouse small intestinal organoids display a complex 3D cell architecture with crypt-like epithelial buds that encompass self-renewing stem cells and differentiated villus-like regions^3^. Upon proliferation, transit-amplifying cells are displaced from the stem cell compartment and differentiate into intestinal cell lineages, including absorptive enterocytes and secretory cells (goblet, Paneth, and enteroendocrine cells), based on gradients of autocrine and paracrine signaling molecules^29^. The differentiated cells are eventually lost and shed into the apical lumen. Therefore, genetic modifications are maintained only if stem cells are targeted (Fig. 1). The progeny of each stem cell then stably propagates the genetic alterations. Because larger organoids contain multiple stem cells, it is worthwhile to consider the cell dynamics during culture. In vivo, each crypt contains ~12–16 dividing stem cells and progressively becomes monoclonal via a process called “neutral drift”^30,31^. During organoid passaging, individual stem cell clones become separated, which can also result in monoclonality. Here, the method of passaging, either as single cells or as larger fragments, influences the rate of this process (Fig. 2). The fact that cells remain coherent in the 3D extracellular matrix (ECM) later assists in obtaining clonal lines by manual “picking” of individual organoids.

Within the epithelium, stem cells are in close contact with other cell types and the ECM, which together constitute the niche environment. In an intact organoid, the cellular context and the surrounding ECM limit the accessibility of stem cells for genetic modification. For this reason, single-cell-dissociated organoids are generally used in most protocols for transgenesis. During enzymatic dissociation, however, cells are vulnerable, and gentle digestion protocols (e.g., the use of Accutase) should be performed to increase outgrowth. Another important requirement is the addition of Rho-kinase inhibitors that effectively block contact loss-induced cell death (anoikis) in epithelial stem cells^3^. The growth conditions have to be adapted to favor the outgrowth of single cells by the addition of niche factors such as Wnt or pharmacological activators of stem cells^22–26,32^. Current protocols aim to expand the proportion of active cycling stem cells before genetic manipulation to later increase the efficacy of colony outgrowth. Optimized recombinant growth factors such as surrogate Wnt agonists help to further increase the success of modification by enhancing single-cell outgrowth and promoting increased long-term self-renewal^33,34^. When organoid systems do not tolerate single-cell outgrowth, intact 3D organoids may be genetically modified^35^. However, with this method, only a fraction of cells in the organoid are targeted, resulting in genetic mosaicism, and homogeneous organoids can only be obtained upon passaging (Fig. 2). Derivation and confirmation of clonal organoid lines can be time-demanding or hampered by a compromised cellular phenotype. Depending on the experimental needs, appropriate methods should be chosen for transgene delivery, organoid propagation, and selection. The following sections provide guidance to select a suitable strategy for successful modification.

## Methods for the delivery and propagation of transgenes in organoids

**Fig. 3:**
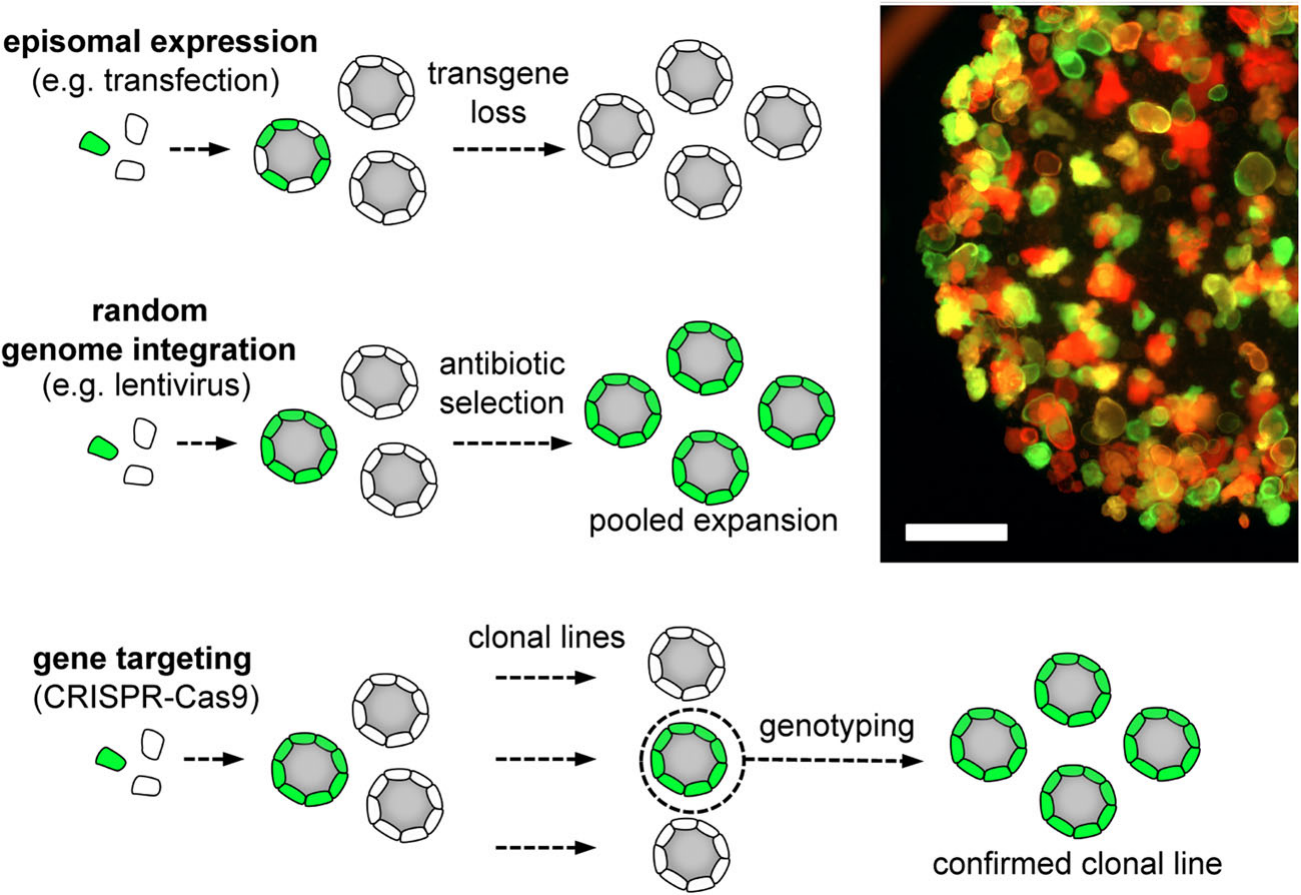
Strategies for the development of genetically modified organoids. Top: Episomal transgene delivery, e.g., by transfection or electroporation of DNA plasmids, only allows transient expression, and organoids show progressive loss of transgene expression. Middle: Stable integration of transgenes, e.g., by lentiviral transduction combined with antibiotic selection, results in a population with homogenous expression. Right: Microscopic image showing the expression of green and red fluorescent lentiviral reporters after selection. Note that most organoids have a single color, indicating clonal outgrowth. Scale bar, 1 mm. Bottom: CRISPR/Cas9 technology allows targeting specific genomic loci. Correctly modified organoid lines can be obtained by the expansion of individual clones and subsequent genotyping.

A variety of protocols are available for the introduction of foreign biomolecules such as DNA, RNA, or proteins into organoids. Transfection using lipophilic reagents results in relatively low efficacy of <10% in mouse small intestinal organoids^22^. Despite this limitation, this method is widely used to introduce small plasmid-based vectors and large bacterial artificial chromosome constructs. Alternatively, electroporation can be performed, and conditions have been described for the transfer of plasmids into human organoids at higher efficacies than achieved by lipofection, ranging between 30 and 70%^23,24,36^. Both methods result in the transient introduction of foreign material, which causes mosaicism of the outgrowing organoids and progressive loss of the transgene (Fig. 3). For this reason, these protocols are particularly suited to introduce enzymes that are only required temporarily, such as Cre recombinase or gRNA plasmids for CRISPR/Cas9 modification (see below). Cotransfection of fluorescent reporter plasmids can help to establish appropriate conditions for gene delivery by allowing image- and fluorescence-activated cell sorting (FACS)-based monitoring of transgene expression. For plasmid-based stable gene delivery, transposon systems such as “PiggyBac” have been successfully used in organoids^23,24^. The transposase enzyme integrates sequences that are inserted between the PiggyBac-inverted terminal repeats into the host genome. Therefore, modification of stem cells allows stable propagation of the transgene but may also cause insertional mutagenesis.

Retro- or lentiviral transduction is a highly efficient method for stable modification of organoids^25,26^. It should be considered that random integration of the virus into the host genome is potentially mutagenic, particularly when high virus titers are used. The viral packaging system determines the tropism and the genetic safety level. In human colon organoids, high transduction efficiencies can be achieved, and lentiviral supernatants may be used without a prior concentration of virus particles^37^. However, the size of the genetic cargo is limited, and large inserts may reduce the titer^38^. Furthermore, the number of integrations per cell can be variable in organoids^39^, and transgene expression may be silenced by epigenetic mechanisms, resulting in heterogeneous expression within the cell population. To circumvent this last problem, vectors should include promoters that are less sensitive to silencing (reported examples are EF1α and PGK)^40,41^ rather than rely on CMV- or LTR-driven transgene expression that can result in mosaicism. In addition, selection cassettes that allow direct enrichment of transgene-expressing cells should be included. For this purpose, resistance genes for antibiotics such as puromycin, blasticidin, or zeocin should optimally be coexpressed with the transgene, e.g., via a P2A or IRES sequence (Fig. 3). Furthermore, fluorescent reporter proteins are very informative for monitoring transgene expression and purifying cells by FACS or by manual picking of organoids from Matrigel. Therefore, homogenous expression can be achieved in an initially heterogeneous organoid population. In addition to constitutive promoters, viral vectors can also be adapted to induce cell-type-specific or ligand-inducible gene expression. Synthetic promoters such as the stem cell Ascl2 reporter (STAR) fragment can be introduced into lentiviral constructs to drive stem cell-specific expression^42^. The most commonly used inducible systems are tetracycline-regulated lentiviral vector systems^43,44^. Lentiviral transduction is often used for the expression of reporter constructs or the introduction of oncogenes^24,45^.

The study of tissue interactions requires even more complex experimental settings, such as organoid cocultures. For cell-type-specific transgene delivery in heterogeneous cell populations, viral vectors with cell-specific tropism may be used^46^. In cocultures, however, single-cell dissociation and antibiotic selection of transgene expression may complicate efficient transduction. Alternatively, cell-type-specific recombinases can be used to control gene expression in cocultures.

## Targeted genetic engineering in 3D organoids

The CRISPR/Cas9 system has allowed the broad application of programmable nucleases for genetic engineering^47^, e.g., to introduce targeted mutations in organoids (Fig. 3). Target specificity is mediated by small guide RNAs (gRNAs) that bind to complementary genomic regions and recruit the Cas9 endonuclease to induce double-strand breaks (DSBs). Error-prone DNA repair by nonhomologous end-joining (NHEJ) then creates insertions or deletions (indels) at the cleavage site, which can be exploited to induce loss of function by frameshift mutations. In the presence of a repair template, DSBs can also be joined by homology-directed repair (HDR), which can be used to insert defined DNA sequences. Depending on the nature and size of the modification, oligonucleotides or plasmid-based targeting vectors are used for HDR. Because HDR has lower efficacy than NHEJ, successful integration of the repair template occurs frequently together with NHEJ-induced indels of the second allele^48–50^.

**Fig. 4:**
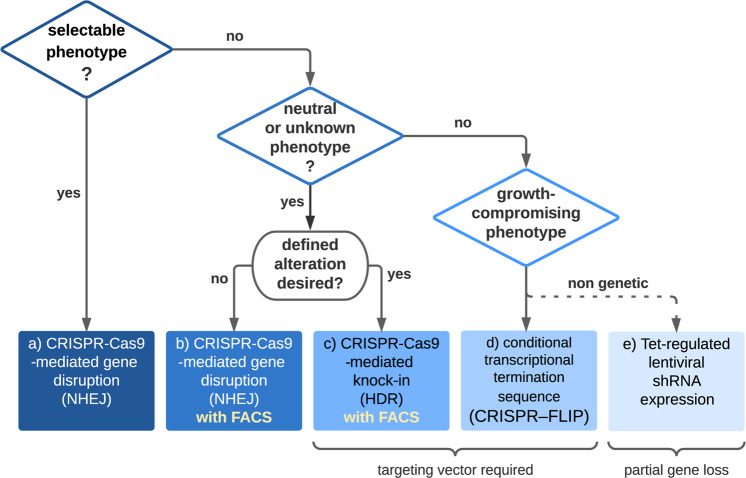
Considerations for successful modification of gene function in organoids. Flowchart depicting key questions that determine which strategies are most effective to obtain a desired transgenic organoid line. A factor is the anticipated phenotype of the gene of interest. Positively selectable traits such as growth advantages or discernable morphologic changes can be used to identify clonal organoids after NHEJ-mediated gene loss (a). For modifications that result in neutral or unknown phenotypes, a pre-enrichment strategy (e.g., by FACS) should be considered (b). If a defined alteration rather than gene loss is desired, classical HDR-mediated knock-in is recommended (c). To address phenotypes that result in compromised growth, inducible strategies, e.g., insertion of a conditional transcriptional termination sequence (d) or by knockdown using Tet-regulated lentiviral shRNA (e), are required. For additional information on the different approaches (a–e), please refer to Table 1.

In many 2D cell culture systems, NHEJ is efficient enough to achieve population-wide gene knockout^47^. This allows direct analysis of cell pools, circumventing the requirement for expanding single-cell clones. However, for loss-of-function studies in organoids, CRISPR/Cas9-induced NHEJ is generally less efficient than in 2D cell culture systems, and characterization of individual clonal lines is recommended to identify correct targeting events. Confirmation of successful biallelic targeting is performed by genomic PCR, bacterial cloning of PCR products, and subsequent Sanger sequencing. Without enrichment strategies, NHEJ-induced frameshift mutations are typically found at efficiencies in the order of 1%, and HDR-mediated introduction of targeted mutations exhibits even lower frequencies^24,45,51^. New bioinformatic tools, such as the “Inference of CRISPR Edits” (ICE) assay^52^, have become very useful to assess modification in organoids. This analysis method allows deconvolution of Sanger sequencing data from mixed cell populations to identify editing outcomes and their frequencies. For phenotypic characterization, incomplete editing complicates the direct analysis of organoid pools after Cas9 administration. Sufficient input material and strategies for genotyping are required to identify and expand correctly modified clones. Furthermore, the study of multicellular organoids is desired to address complex phenotypes such as epithelial barrier function or cell-type-specific differentiation. However, in cases where genetic manipulation compromises cell proliferation or stem cell function, an inducible approach becomes necessary. Consequently, based on the nature of the desired genetic manipulation and the expected phenotype, a suitable experimental strategy should be selected to assure experimental success. In the following section, we compare different scenarios and suggest available strategies for genetic modification of organoids that also consider workload and scalability. A flowchart summarizing the parameters that define the optimal method is shown in Fig. 4, and detailed information is provided in Table 1.

**Table 1:** Comparison of available strategies for genetic manipulation of gene function in organoids.

Anticipated phenotype	Experimental strategy	Pros and cons	Recommended best practices	References
(a) Selectable traits, e.g., growth-factor dependence or morphologic change	Cas9-mediated gene disruption	+ high efficiency due to phenotypic selection − variable NHEJ repair outcomes	Allows clonal expansion and genotyping of several independent organoid lines.	^24,45,51^
(b) Neutral or unknown phenotype	Cas9-mediated gene disruption combined with FACS purification	+ flexible and scalable − requires optimization of Cas9/gRNA delivery − variable NHEJ repair outcomes	Clonal expansion and genotyping of a moderate number of organoid lines is required.	^53,54^
(c) Neutral or unknown phenotype	Cas9-directed HDR using targeting vectors	+ generates defined genetic changes − low efficacy of HDR − cloning of targeting vector and genotyping are labor-intensive − the second allele is often deleted	Clonal expansion and genotyping of a high number of organoid lines are required. Improved efficacy by FACS pre-enrichment and Cas9 RNP.	^36,51,56,57^
(d) Growth-compromising or unknown phenotype	HDR-mediated insertion of a conditional transcriptional termination sequence (CRISPR–FLIP)	+ efficient method for generation of conditional alleles + allows antibiotic selection of correctly targeted cells − requires cloning of targeting vector and introduction of Cre	Clonal expansion and genotyping of a moderate number of organoid lines are required.	^58^
(e) Growth compromising or unknown phenotype	Tet-regulated lentiviral shRNA expression	+ allows temporal control of gene expression + allows pooled analysis after antibiotic selection − allows reduction of gene function rather than gene loss	Knockdown efficiency should be validated. Several shRNAs and control shRNAs should be studied.	^43,44^

Without selection, transient delivery of CRISPR/Cas9 reagents only permits inefficient modification. Despite this limitation, organoids are particularly suited for the engineering of genetic traits that provide selectable growth advantages (Table 1). Suitable phenotypes for “positive selection” are independence from growth factors (e.g., Wnt or EGF) or resistance to growth-inhibiting factors (e.g., TGFβ or P53 stabilization by Nutlin-3)^24,45^. In addition, morphological parameters such as organoid swelling after forskolin treatment^51^ can be used to identify correctly targeted organoids for the establishment of clonal lines. The availability of a stringent phenotype greatly assists the identification of correct organoids with targeting frequencies of up to 100%. Thus, with sufficient starting material, even rare events, e.g., simultaneous knockout of multiple genes or introduction of defined point mutations by CRISPR-mediated HDR, can be identified^24,45^. This strategy has been very valuable for engineering recurrent oncogene and tumor suppressor mutations in colon organoids and thereby stimulating tumor progression.

When phenotypes are neutral and do not allow selection, alternative strategies are required to enrich modified cells (Table 1). Fluorescent reporters can be cotransfected or introduced into lentiviral vectors to pre-enrich modified cells by FACS. However, this approach guarantees the proper introduction of only the CRISPR/Cas9 components and not the stochastic gene targeting itself; therefore, multiple clones should be expanded and genotyped individually. The efficient modification requires a sufficient expression level of Cas9, which can either be introduced in a stable fashion^37^ or transfected as a ribonucleoprotein (RNP) complex of a gRNA and purified Cas9 protein^36^. Cas9 RNP delivery has several advantages, including immediate activity and reduced off-target modification. In a recent study, organoids from Rosa26-Cas9 mice were transfected with in vitro transcribed gRNAs together with GFP mRNA^53^. In that study, FACS-based pre-enrichment allowed successful homozygous knockout of eight candidate genes to study the role in the differentiation of enteroendocrine cells. For each candidate, the authors characterized 16 independent organoid clones, demonstrating a powerful protocol to investigate gene function on an intermediate scale. Another study reported the use of NHEJ for locus-specific insertion of reporter constructs in human organoids from the intestine, liver, and bile ducts^54,55^. The CRISPR-HOT method is powerful for targeting endogenous genes that show cell-type-specific expression or that encode proteins with defined subcellular localization. The microscopic inspection then allows the generation of multicolor organoid lines, e.g., for live imaging studies.

For HDR-mediated introduction of defined mutations, coadministration of fluorescent reporters with CRISPR/Cas9 reagents is not sufficient for pre-enrichment of cells. Instead, classical targeting vectors that contain homology arms for targeted insertion of antibiotic resistance cassettes and/or fluorescent reporters should be used (Table 1). After antibiotic selection, correct repair of the mutant cystic fibrosis transmembrane conductor receptor (*CFTR*) locus was achieved in ~20% of clones^51^, providing a proof of concept for CRISPR/Cas9-mediated gene therapy. Two recent studies showed successful reporter knock-in of GFP into the human *LGR5* locus in colorectal cancer organoids. In the first study, antibiotic selection and picking of GFP-positive clones were used to enrich for clones with correct integration, which could be subsequently confirmed by genotyping 5–10 independent clones^56^. In the second study, after nucleofection, Cas9 and gRNA expression was first pre-enriched by FACS. In the resulting colonies, ~10% of cells were GFP-positive, indicating proper integration of the promoterless HDR template into the *LGR5* locus, a finding that was confirmed by genotyping^57^. To avoid interference of the inserted construct with target gene expression, the selection cassette may need to be removed later, e.g., by transient transfection with recombinase using a Cre/loxP strategy. Combined with the requirements for individual targeting vectors and careful optimization of organoid selection, HDR-mediated engineering is a relatively demanding technique. In a recent study, the use of a Cas9/gRNA RNP combined with FACS enrichment of GFP knock-in allowed remarkable efficiencies of up to 60% in human fetal lung organoids^36^.

The low efficiency of the Cas9 system becomes problematic when the desired modifications have a negative effect on cell growth. In the case of growth-compromising or lethal phenotypes, fluorescence-based preselection of clones is not sufficient, because the fraction of transfected but unmodified cells overgrow the successfully targeted cells. Instead, inducible methods are required (Table 1), and one powerful strategy, CRISPR–FLIP, has recently been described^58^. The technique is based on the HDR-mediated insertion of a Cre/loxP invertible cassette into an intronic region of a target gene. The original non-mutagenic orientation of the cassette enables selection by antibiotic resistance and thereby enrichment of clones with correct integration of the targeting vector. Upon inversion of the cassette by Cre recombinase, an artificial splice acceptor site, and polyadenylation signal disrupt target gene expression. The second allele is usually lost via constitutive NHEJ-mediated repair. This inversion strategy is highly efficient, and modified cassettes also allow reversible inactivation using a second recombinase, as shown for the ablation and re-expression of β-catenin in mouse embryonic stem cells. Generally, the Cre-Lox recombination system provides a very efficient technology to induce specific genetic changes. In particular, mouse organoids can be easily generated from the many available strains harboring conditional alleles. Recombination can be induced by lentiviral transduction of Cre^59^, transfection of a Cre plasmid, or protein^60^. For temporal control, organoids can be established from mice expressing ligand-inducible Cre, which allows deletion of floxed alleles or induction of lentiviral transgenes (using LoxP-containing cassettes) upon incubation with the activating ligand (e.g., 4-hydroxytamoxifen), e.g., to dynamically study the role of Notch pathway activity on goblet cell differentiation^25^.

RNA interference is another useful strategy to study loss-of-function phenotypes in organoids (Table 1). Population-wide expression of lentiviral short hairpin RNAs (shRNAs) can be assured by antibiotic selection. This approach facilitates direct analysis of organoid pools after transduction without the need to establish clonal organoid lines^43,44^, increasing the experimental scalability. Furthermore, the availability of tetracycline-inducible shRNA lentiviral vectors allows precise control of gene expression in larger organoids. This method has been used to study the influence of the chromatin modulator Hmga1 on intestinal stem cell regulation^43^ and the mechanisms of resistance to Wnt pathway inhibition^44^. In contrast to genetic manipulation, RNA interference may only reduce and not completely ablate gene function; however, for some experiments, reduction of gene function rather than gene loss may be advantageous (e.g., studies on haploinsufficient genes). The knockdown efficiency should be validated at the mRNA and protein levels.

## Strategies to prevent off-target modification

The introduction of nucleases carries the risk of unwanted modifications. To date, no next-generation-based sequencing has been performed to identify all possible alterations in an unbiased fashion. Instead, based on similarity to the target site, predicted off-target locations have been analyzed individually, which has shown the high fidelity of the CRISPR/Cas9 system in clonal organoid lines^24,51,57^. However, during experimental design, several factors should be considered to avoid confounding effects. The transient activity of CRISPR/Cas9 is recommended to reduce off-target modifications that may otherwise accumulate over time. Administration of Cas9 RNP allows direct and highly specific editing in primary cells, including organoids^36,61^. Furthermore, analysis of clonal organoids requires caution because single alterations can become fully penetrant (Fig. 2), and off-target editing, as well as spontaneous mutations, can thus have a strong impact. Analysis of pools of independently modified organoids can help to reduce the influence of bystander mutations. However, when this analysis is not possible, e.g., due to insufficient editing in the pooled population, analysis of several independently genotyped organoid clones is recommended. In addition, experimental validation using alternative gRNA sequences should be considered. Engineered variants of the Cas9 nuclease, e.g., eSp-Cas9^62^ or HiFi Cas9^63^, have been reported to reduce off-target modification and have been successfully used in organoids^36^. To reduce unwanted DSBs, Cas9 nickases can alternatively be used in combination with gRNA pairs^64^. In knock-in experiments, the targeting vector may also integrate at off-target sites or in a random fashion. Therefore, correct transgene integration should always be verified by sequencing PCR amplicons using primers that bind within the insert and in flanking genomic regions. Because HDR-mediated knock-in generally occurs in a monoallelic fashion, the second allele should be characterized to identify the occurrence of NHEJ-mediated mutagenesis.

## Further applications using CRISPR/Cas9 to study gene function in organoids

**Fig. 5:**
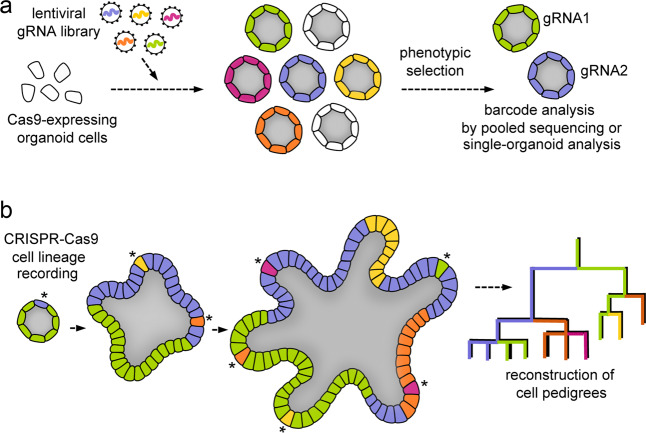
Recent experimental approaches using CRISPR/Cas9 in organoids. **a** Unbiased genetic screening in organoids after transduction with lentiviral gRNA libraries. After selection (e.g., for a growth advantage), the gRNA barcode distribution is analyzed by next-generation sequencing in a pooled organoid population or in individual organoid clones to identify genetic mediators. **b** Time-resolved lineage tracing using CRISPR/Cas9 techniques. The principle is based on the NHEJ-mediated introduction of “genetic scars” (asterisks) at neutral genomic positions that can be used to infer the phylogenetic history of cells.

Lentiviral CRISPR/Cas9 library screens have become a very useful method to study the function of unknown genes in cell lines. We and others have recently developed protocols for screening 3D organoids that take into account specific requirements with respect to scale and growth dynamics^37,39^. In both studies, organoids were transduced with a lentiviral gRNA library and were then subjected to phenotypic selection (Fig. 5a). Positively selected organoids were studied to identify genes that confer resistance to TGF-β-induced cell death. In addition, tumor suppressors that promote subcutaneous tumor growth were analyzed after organoid transplantation in mice. The greatest challenge was to attain sufficient library coverage, which is hampered by the cost of culture components. For this purpose, we opted for focused libraries that contained 2000–3000 individual gRNAs. At this scale, organoid pools can be reliably analyzed by next-generation-based sequencing of gRNA barcodes^37^. In contrast, Ringel et al.^39^ used a genome-wide library including >70,000 gRNAs, which posed a challenge for reaching coverage for a robust pooled readout. Instead, organoids were sequenced individually, which allowed the identification of novel TGF-β resistance mediators. In addition, this method allowed the exclusion of potential false-positive hits that were caused by simultaneous integration of multiple lentiviral vectors. During phenotypic selection, the organoid population undergoes a progressive drift that can cause expansion or loss of individual clones. This drift carries the risk that false-positive hits acquire a dominating effect in a pooled analysis of gRNA barcodes. To circumvent this issue, we combined unique molecular identifiers (UMIs) with each gRNA lentivirus to monitor individual organoid clones after phenotypic selection^37^. UMI libraries can allow the determination of how often a certain gRNA causes organoid growth (incidence) instead of only the abundance of the gRNA, which is strongly influenced by the variable organoid size. This increased resolution allows the removal of false-positive outlier clones, improving the robustness of organoid-based screens. However, a side-by-side comparison with experiments in classical 2D cell lines revealed important challenges for CRISPR experiments in organoids^37^. Although some gRNAs were highly efficient, a considerable fraction showed reduced activity in organoids, indicating that the current gRNA design rules are not optimal for predicting activity. This impairment could in part be linked to the WT status of *P53* in organoids, which can lead to toxic stress after the generation of CRISPR/Cas9-induced DSBs^65^. Future experiments would greatly benefit from the availability of gRNA reagents with validated activity in organoids to generate more effective libraries for 3D screens.

While clonal tracing using lentiviral UMI sequences can be informative to describe the composition of the organoid population, this method lacks the resolution to monitor dynamic processes such as stepwise differentiation into cell lineages. Recently, several technologies that also allow time-controlled or even continuous lineage tracing have been described^66–69^ (Fig. 5b). To this end, sgRNAs that target either their own genomic sequence or neutral reporter loci are introduced, and Cas9 activity and NHEJ-mediated repair are then induced. This strategy leads to the acquisition of characteristic indel mutations that can serve as cellular barcodes that are inherited by a cell’s progeny. Next-generation sequencing then allows to trace these barcodes in many cells in parallel to generate cellular pedigrees that reflect the developmental history of the population. Cell identity information can be integrated with single-cell RNA sequencing data, providing detailed information on differentiation processes in different organoids, e.g., brain organoids^70^.

A number of developments may further expand the experimental potential of programmable nucleases in organoids. Protein engineering has led to more accurate Cas9 variants^62,63,71^ and inducible enzymes^72–74^. Furthermore, CRISPR/Cas9 systems from other organisms^75,76^, as well as alternative nucleases^77,78^, have been described. Beyond genetic engineering, the targeting capabilities of Cas9 proteins have been exploited for epigenetic manipulation. Gene expression can be manipulated by CRISPR interference (CRISPRi) or CRISPR activation (CRISPRa) using nuclease-defective Cas9 variants that are fused to transcriptional regulators^79^. The resulting gene perturbation is reversible and independent of stochastic DNA repair mechanisms and the associated potential genotoxic stress. Therefore, CRISPRi/a strategies may permit more direct control of gene expression, which may be beneficial for future screening applications in organoids or as an alternative to shRNA-mediated RNA interference (Table 1). Fusion of nuclease-defective Cas9 with DNA editing enzymes has led to the development of enzymes that can specifically catalyze the conversion of single nucleotides (“base editors”)^80^. This strategy allows the generation of specific alterations and has been successfully used in organoids to induce oncogenic mutations in the mouse *Apc* and *Pik3ca* genes^81^ and to correct pathogenic mutations at the human *CFTR* locus^82^. However, because base editing depends on proximity to a gRNA target site and induces specific substitutions, not all desired alterations are experimentally feasible. Another strategy to introduce targeted mutations, “prime editing”, has recently been described. The key components of this strategy are the “prime editor” (PE), a fusion protein of Cas9 nickase and reverse transcriptase^83^, and the “prime editing gRNA” (pegRNA), which combines locus-specific binding with its function as a template for reverse transcription. After DNA binding and nicking, the PE-pegRNA complex mediates the first-strand synthesis followed by precise integration and replacement of the edited strand in the host genome. The system allows the introduction of defined insertions, deletions, and point mutations and is, therefore, more flexible than base editing, which can only introduce specific base conversions. Prime editing and base editing have the shared advantages of being independent of potentially harmful double-strand breaks and not requiring a donor DNA template. In human intestinal organoids, prime editing has low off-target activity and was successfully used to repair mutations at the *CFTR* or *DGAT1* loci or to introduce diverse pathogenic mutations (e.g., in the *TP53* or *CTNNB1* genes)^50,84^. Comparative analysis in organoids showed that base editing, when possible, is more reliable and efficient than prime editing. However, the more versatile prime editing was found to be superior to HDR-mediated mutagenesis^50,84^.

## Concluding remarks

Applications for adult stem cell-based organoids have rapidly evolved in recent years and have greatly expanded the possibilities for genetic modification. These developments have great potential to improve our molecular and cellular understanding of human tissue biology. However, some organoid-specific challenges remain unsolved. The limited scalability and reduced efficacy of the CRISPR/Cas9 systems restrict experimental design in organoid systems compared to classical 2D cell line systems. To overcome these challenges, recent protocols aim to optimize transgene delivery and gene editing efficiency in organoids^36,85,86^. Other developments aim to improve the survival of single cells^33,34^ or the selection of correctly targeted cells^87^. Concepts similar to those discussed in this review may be useful in other stem cell-driven organoid systems using appropriate culture conditions and adapted protocols for transgenesis^88–90^. In organoids derived from induced pluripotent stem cells (iPSCs), transgenic modification can be efficiently introduced in 2D systems at the iPSC stage^91,92^. To study phenotypic consequences at later stages of differentiation, inducible systems are used for temporal control. For example, differentiation into enteroendocrine cells was induced in intestinal organoids derived from human pluripotent stem cells (hPSCs) harboring an inducible NEUROG3 expression cassette^93,94^.

New features, such as clonal tracing^66–69^ and techniques that allow more direct gene perturbations, such as CRISPRi/a^79^ and prime editing^83^, could help to further improve the flexibility of genetic manipulation of organoids in the future. In addition, the development of more cost-effective synthetic ECMs or optimization of library design^95^ may increase the possibilities for genome-wide screening. In complex structures, such as multicellular organoids, single-cell omics readouts will become increasingly informative. scRNA sequencing in combination with gene perturbation may allow deep phenotypic analysis^96,97^. Furthermore, the combination of CRISPR barcoding with single-cell RNA sequencing^66–69^ will be informative for experimentally recording differentiation trajectories. Image-based readouts and computational methods will be used to assign complex phenotypes^98^. In summary, the multicellularity and phenotypic plasticity of organoids have prompted the development of unprecedented experimental models to study physiologic and pathologic processes in human cells that will be further enhanced with the development of appropriate genetic tools.
